# Derivation and validation of a computable phenotype for acute decompensated heart failure in hospitalized patients

**DOI:** 10.1186/s12911-020-1092-5

**Published:** 2020-05-07

**Authors:** Rahul Kashyap, Kumar Sarvottam, Gregory A. Wilson, Jacob C. Jentzer, Mohamed O. Seisa, Kianoush B. Kashani

**Affiliations:** 1 Multidisciplinary Epidemiological and Translational Research in Intensive Care, Mayo Clinic, Rochester, MN USA; 2 Department of Anesthesiology & Perioperative Medicine, Mayo Clinic, 200 First Street SW, Rochester, MN 55905 USA; 3 Division of Nephrology and Hypertension, Department of Internal Medicine, Mayo Clinic, Rochester, MN USA; 4 Pulmonary Critical Care Division, Einstein Medical Center, Philadelphia, PA USA; 5 Department of Cardiovascular Diseases, Mayo Clinic, Rochester, MN USA; 6 Division of Pulmonary and Critical Care Medicine, Department of Internal Medicine, Mayo Clinic, Rochester, MN USA

**Keywords:** Acute decompensated heart failure, Acute heart failure, ADHF, Electronic algorithm, ICD-9

## Abstract

**Background:**

With higher adoption of electronic health records at health-care centers, electronic search algorithms (computable phenotype) for identifying acute decompensated heart failure (ADHF) among hospitalized patients can be an invaluable tool to enhance data abstraction accuracy and efficacy in order to improve clinical research accrual and patient centered outcomes. We aimed to derive and validate a computable phenotype for ADHF in hospitalized patients.

**Methods:**

We screened 256, 443 eligible (age > 18 years and with prior research authorization) individuals who were admitted to Mayo Clinic Hospital in Rochester, MN, from January 1, 2006, through December 31, 2014. Using a randomly selected derivation cohort of 938 patients, several iterations of a free-text electronic search were developed and refined. The computable phenotype was subsequently validated in an independent cohort 100 patients. The sensitivity and specificity of the computable phenotype were compared to the gold standard (expert review of charts) and International Classification of Diseases-9 (ICD-9) codes for Acute Heart Failure.

**Results:**

In the derivation cohort, the computable phenotype achieved a sensitivity of 97.5%, and specificity of 100%, whereas ICD-9 codes for Acute Heart Failure achieved a sensitivity of 47.5% and specificity of 96.7%. When all Heart Failure codes (ICD-9) were used, sensitivity and specificity were 97.5 and 86.6%, respectively. In the validation cohort, the sensitivity and specificity of the computable phenotype were 100 and 98.5%. The sensitivity and specificity for the ICD-9 codes (Acute Heart Failure) were 42 and 98.5%. Upon use of all Heart Failure codes (ICD-9), sensitivity and specificity were 96.8 and 91.3%.

**Conclusions:**

Our results suggest that using computable phenotype to ascertain ADHF from the clinical notes contained within the electronic medical record are feasible and reliable. Our computable phenotype outperformed ICD-9 codes for the detection of ADHF.

## Background

The utility of electronic health records (EHRs) has been increased in past decade and the size of available health information for clinical and epidemiologic research has rapidly stretched [1, 2]. This brings new hurdles for current methodology, such as the inability to manually review sufficient amounts of data in a reasonable time period, the use of inadequate search strategies to review the EHR, and the reliance on the variable accuracy of ICD-9 (International Classification of Diseases, Ninth Revision) billing codes [3–5].

Newer computable phenotypes (automated electronic search strategies) have been created to facilitate data collection. For instance, search algorithms have been successfully developed to identify postoperative cardiovascular and thromboembolic complications [6], Charlson comorbidity index [7], risk factors for acute lung injury [8], initiation of emergent intubations in the intensive care unit (ICU) [9], extubation time in the ICU [10], chronic co-morbidity phenotypes from the EHR, and genomics studies [11]. Recently, for better provider decision making for sepsis care, supervised machine learning has been deployed as two-step machine-human interface [12]. These studies have all demonstrated that electronic searches can achieve sensitivities and specificities greater than 90% when compared to manual search efforts. Additionally, a previous study demonstrated portability of such electronic search tools, potentially allowing for application of search algorithms at external institutions [13]. However, there is limited literature on automation for identifying acute decompensated heart failure (ADHF) and the effectiveness of such methodology, specifically, when compared to the manual chart review of a prospectively collected electronic database.

The incidence of heart failure (HF) is growing in the past decades; currently, it is reported that it impacts more than 20 million people around the world and more than 5.5 million individuals in the United States, being the top discharge diagnosis among medicare beneficiaries [14, 15]. As per Joseph et al. the ADHF’s definition is “the sudden or gradual onset of the signs or symptoms of heart failure requiring unplanned office visits, emergency room visits, or hospitalization”. One of the omnipresent feature of ADHF is pulmonary and systemic congestion due to increased left- and right-heart filling pressures, which is neutral to any exacerbation mechanism [2, 16]. Hospitalization for ADHF is a powerful predictor of readmission and post-discharge death in patients with chronic HF, with mortality rates as high as 20% after discharge [17, 18]. Regardless of the etiology, inpatient treatment for ADHF portends a worsening prognosis [19]. Despite this, there is no previously defined tool to identify heart failure from EHR accurately and efficiently.

The primary aim of this study was to develop and validate a computable phenotype to detect the presence of ADHF in a retrospective cohort of patients admitted to a tertiary care center (Mayo Clinic Hospitals in Rochester, MN). Additionally, we wanted to compare the performance of this computable phenotype to ICD-9 code search and gold standard (expert manual reviews). We hypothesized the automated computable phenotype would be as good as the gold standard and outperform search strategies using the ICD-9 Code system.

## Methods

### Study population

**Fig. 1:**
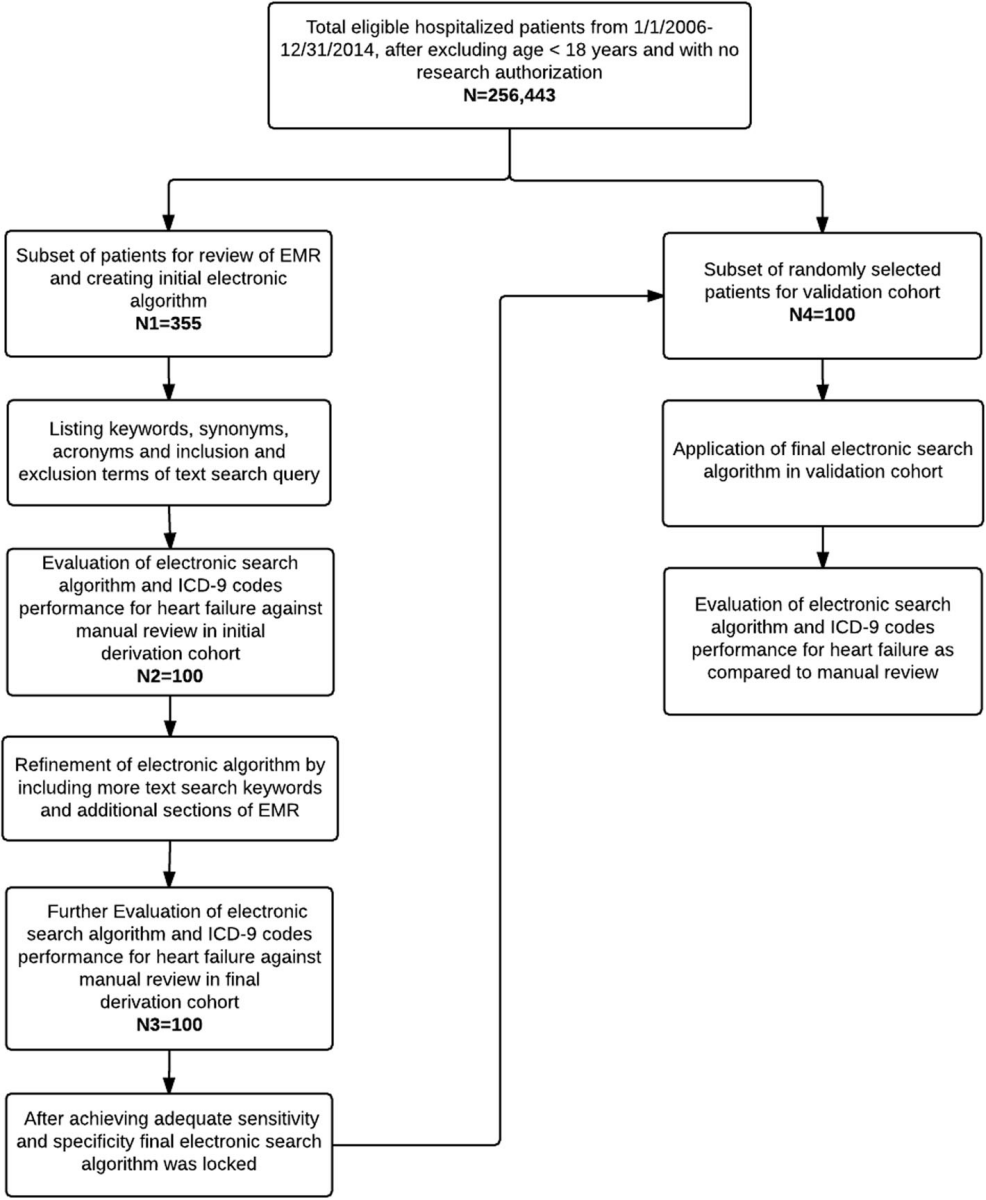
Flowchart of Included Patients in Derivation and Validation Cohorts

The study was approved by the Mayo Clinic Institutional Review Board in year 2015 for the use of existing medical records of patients who were admitted to Mayo Clinic, Rochester, MN from January 1, 2006, through December 31, 2014. The derivation and validation subsets were randomly selected from a cohort of 256,443 eligible adult patients’ (≥18 years of age) with prior research authorization (Fig. 1).

### Identification of study population

Patient hospitalization data was extracted from the UDP using an ACE hospital admission query. A total of 473,146 hospital admits in 314,988 patients were found during the study period listed above. From the patient number of 314,988 there were 44,867 patients excluded for age less than 18 years, 13,678 patients excluded for no research authorization in accordance with the Minnesota Health Records Act (Minnesota Statue 144.291–144.298) that states only patients who consented research authorization of their medical record data can be included for study, and 256,443 patients included ad total eligible patients.

### Data extraction strategies

For the purpose of algorithm creation, the EHR of a subset (N1 = 355) of patients, derived from the total eligible patients, were manually reviewed to identify keywords and inclusion & exclusion terms for ADHF (see Additional file 1 for added explanation of algorithm creation). The performance of this algorithm was evaluated at multiple steps of derivation using an initial derivation cohort of 100 patients (N2). With the goal to achieve sensitivity of more than 95%, the automated search algorithm was continuously refined with additional free text search terms, inclusion and exclusion keywords, and the inclusion of more datasets in EHR. After > 95% sensitivity and specificity were achieved and tested on a final derivation cohort of another 100 patients (N3), the algorithm was then validated in an independent cohort of 100 patients (N4) (see Fig. 1 for a flowchart of the extraction strategy).

### Manual data extraction strategy

Manual review of patient records is the traditional method for data abstraction; there is no established gold standard to validate ADHF during hospital stay based on the EHR. In this study, two investigators- co-authors (KS and MS) independently reviewed the EHR of 355 patients to initiate the algorithm process and refine it in the derivation steps to identify ADHF in its various synonyms and abbreviations within pre-specified EHR sections (For the flowchart of extraction strategy, please see Fig. 1). The manual reviewers were not involved in the development or utilization of the automated electronic search strategy and were blinded to the results from the automated electronic note search strategy. Then subsequent cohorts of 100 and 100 random patients chart were reviewed, thus making the 3 separate derivations cohorts of total 555 patients. Once the algorithms were finalized, the manual chart review was done for another 100 random patients, bringing the whole cohort to 655 patients.

### Automated electronic data extraction strategy

This retrospective study used information from the United Data Platform (UDP), the clinical data repository for Mayo Clinic. The UDP is an exhaustive clinical data warehouse that stores patient demographic characteristics and diagnoses, and hospital, laboratory, clinical, and pathologic data gathered from various clinical and hospital source systems within the institution. We used the Advanced Cohort Explorer (ACE) toolset to access the data contained within the UDP. The ACE can search for demographic characteristics, clinical data, hospital admissions information, diagnosis codes, procedure codes, laboratory test results, flow sheet data, pathology reports, and genetic data. The ACE provides a unique free text search strategy by which researchers can quickly search for slected words or groups of words in the EHR system.

To find patients with ADHF we first had to find patients with any heart failure. To do this a study author adept at building ACE electronic search queries constructed a query using ICD9 codes for heart failure (Listed in Additional file 3). This query was then run on a random sample of 1000 study patients obtained from the hospital admit query. Manual interrogation of clinical notes of the ICD9 query ‘hits’ was done in first 355 patients to determine how ADHF was documented in the patient’s medical record. From this manual interrogation of the clinical notes of the ICD9 ‘hits’ a clinical note text query was created.

The ACE is based on Boolean logic to create free text searches. To prepare a query, we entered all the synonyms, abbreviations, and medical acronyms for ADHF. Also to make the computable phenotype more specific we developed an extensive list of exclusion terms. For example, diagnoses that had the following words associated with them were excluded: “prior,” “rule out,” or “negative for” (for a complete list of inclusion and exclusion terms, see Additional file 1). To establish a more uniform methodology and minimize the number of false-positive results, the application of the automated algorithm to note searches was restricted to the Sections- *“Diagnosis- Principal Diagnosis, - Secondary Diagnoses,- Chief Complaint/Reason for Visit,- Brief Hospital Course.”* The computable phenotype was continuously refined through an iterative process of review of mismatches between the automated electronic search and the manual chart review. Every time a discrepancy between the electronic search and the manual search was identified, the search strategy for inclusion-exclusion criteria were updated and re-examined on the derivation cohort. After multiple iterations, the sensitivity and specificity for ADHF detection improved to greater than 95%, at which point the algorithm was finalized and applied to the validation cohort.

### ICD-9–based data extraction

Acute HF and acute-on-chronic HF (systolic, diastolic or both) have individual specific ICD9 codes. However, the sensitivity and specificity of these codes remains in question. ADHF does encompass the acute HF conditions listed as HF 428 codes (Additional file 2). Because the majority of patients with in-hospital ADHF did have HF ICD-9 coding, albeit not specific to one subcategory, the computable phenotype for ADHF was eventually compared to codes for acute HF (Additional file 2) and also to all codes for HF within the ICD 9.

### Statistical analyses

Sensitivity and specificity of both the computable phenotype and ICD-9 code search were calculated based on comparisons of the test results to the results of manual data abstraction (reference standard) for both the derivation and validation cohorts using JMP statistical software (JMP®, Version 10.0. SAS Institute Inc. Cary, NC). Positive or negative predictive values were not calculated because of their dependence on the incidence rate within the cohort. The 95% confidence intervals were calculated using an exact test for proportions. The computable phenotype was refined over the course of several iterations. The sensitivity and specificity of the search in the derivation cohort were calculated for both the initial and final iteration of the computable phenotype.

Here are the formula for sensitivity *[Sensitivity = true positives/(true positive + false negative)]* and specificity *[Specificity = true negatives/(true negative + false positives)]* (Table 1).

**Table 1:** The 2 by 2 table for calculation of sensitivity and specificity

		Reference standard	
		Positive	Negative
Computer	Positive	True positive	False positive
	Negative	False negative	True negative

## Results

### Performance of computable phenotype as compared to manual review

Initially, the computable phenotype automated electronic ADHF search strategy (eADHF) achieved a sensitivity of 89.4% and specificity of 81% for ADHF in the derivation cohort when analyzed against a manual review in the initial derivation cohort (N2). After several revisions, the eADHF was tested in final derivation cohort (N3), which achieved the sensitivity and specificity of 97.5 and 100% respectively (Table 2). When this finalized eADHF was applied to the validation cohort of 100 patients, the sensitivity and specificity were 100 and 98.6% (Table 3).

**Table 2:** Derivation Cohort- eADHF and ICD-9 heart Failure Code Performance Against Reference Standard

	eADHF vs. reference standard		ICD-9 (acute HF) vs. reference standard		ICD-9 (all HF) vs. reference standard	
	Sensitivity	Specificity	Sensitivity	Specificity	Sensitivity	Specificity
Derivation-Initial (N2=100)	89.4	81	53	95.6	95.5	79.4
Derivation-Final (N3=100)	97.5	100	47.5	96.7	97.5	86.7

**Table 3:** Validation Cohort- eADHF and ICD-9 heart Failure Code Performance Against Reference Standard

	eADHF vs. reference standard		ICD-9 (acute HF) vs. reference standard		ICD-9 (all HF) vs. reference standard	
	Sensitivity	Specificity	Sensitivity	Specificity	Sensitivity	Specificity
Validation (N4=100)	100	98.6	42	98.6	96.7	91.3

### Performance of ‘acute’ heart failure codes of ICD 9 as compared to manual review

In the initial derivation cohort (N2), the sensitivity and specificity of ICD-9 Acute Heart failure codes as compared to manual review was 53 and 95.6%, respectively. In the final derivation cohort (N3) the sensitivity and specificity were 47.5 and 96.7%, respectively (Table 2). In the validation cohort (N4) the sensitivity and specificity were 42 and 98.6%, respectively (Table 3).

### Performance of ‘all’ heart failure codes of ICD 9 as compared to manual review

In the initial derivation cohort (N2), the sensitivity and specificity of ICD-9 All Heart failure codes as compared to manual review was 95.5 and 79.4%, respectively. In the final derivation cohort (N3), the sensitivity and specificity were 97.5 and 86.7%, respectively. In the validation cohort (N4) the sensitivity and specificity were 96.7 and 91.3%, respectively.

### The performance of acute heart failure codes (ICD 9) compared to (eADHF)

In both the final derivation and validation cohort, the eADHF outperformed ICD-9 codes for heart failure. In the final derivation cohort, sensitivity and specificity of the acute codes for ICD-9 heart failure as compared to eADHF was 46.2 and 95.1%, respectively. In the validation cohort sensitivity and specificity of the acute codes for ICD 9 heart failure as compared to eADHF was 43.8 and 100%, respectively (Table 4).

**Table 4:** Derivation and Validation Cohort- ICD-9 Heart Failure Code compared to eADHF

	ICD-9 (acute HF) vs. reference standard		ICD-9 (all HF) vs. reference standard	
	Sensitivity	Specificity	Sensitivity	Specificity
Derivation-Initial (N2=100)	48.7	98.3	93.4	89.7
Derivation-Final (N3=100)	46.2	95.1	97.4	85.3
Validation (N4=100)	43.8	100	96.8	92.7

### The performance of all heart failure codes (ICD 9) compared to eADHF

In both the final derivation and validation cohort, the eADHF outperformed ICD-9 codes for heart failure. In the final derivation cohort sensitivity and specificity of ALL codes for ICD-9 heart failure as compared to the eADHF was 97.4 and 85.3%, respectively. In the validation cohort sensitivity and specificity of ALL codes for ICD-9 heart failure as compared to the eADHF was 96.8 and 92.7%, respectively (Table 4).

## Discussion

The present study compared two independent methodologies for identifying patients with ADHF and demonstrated that the sensitivity and specificity of the computable phenotype could approach 100%. These findings further corroborate previously published studies showing that the use of automated search strategies produced very accurate results that were agreeable with those produced by manual review methods [3, 8, 9, 11].

The previous studies comparing the use of electronic free-text search algorithms applied to the EHR versus administrative ICD-9 data codes to identify ADHF had variable success [20–23]. This study solidifies the advantage of electronically searching the EHR (compared to other electronic search strategies such as ICD-9 data codes) and demonstrates that this method can be as accurate as experts chart review in prospectively collected electronic databases across institutions.

In this era of medical informatics, our approach has numerous benefits. The computable phenotype could substantially decrease human hours spent and human errors reviewing medical charts for research studies requiring ADHF information but also provides consistent results. Furthermore, ACE, which was used in this study, is applicable to any EHR database system. This search algorithm was developed using free text and natural language processing strategies that broadly reflect clinical practice (Additional files 1 and 2), and it does not rely on any complex coded electronic information, specialized image tests, or any other diagnostic modalities.

To minimize Type 1 error and maximize our chances of capturing 100% of events, a broad spectrum of inclusion and exclusion terms (Additional files 1 and 2) were incorporated in this study, making provision for the potential differences in semantics and documentation language across various institutions, departments, and data structures. The usability of such search algorithms has been demonstrated in the past across multiple health-care institutions and EHRs with minimal algorithm optimization for institution-pertinent needs [13]. It increases the efficiency of clinical and translational research. Further studies are needed to evaluate if it can indirectly enhance the quality of patient care by providing information about risk factors for adverse outcomes of interest. For instance, information regarding ADHF may permit investigation of risk factors and adverse outcomes associated with ADHF. In this case, the strategy may accelerate any clinical research that may involve analyzing other adverse events such as cardiorenal syndrome, complications of ADHF, and other prognostic impacts as well. It has potential for the surveillance of admitted patients with ADHF. This could be associated with a Clinical Decision Support System that could be available to the clinicians to optimize the care of these patients.

There are several limitations of this study that we would like to point out. The data quality is as good as the source hospital database. Any incorrect data entry or missing data points or corrupted database may cause some inaccuracies, but this limitation likely accounts for a minority of patients in the database [24, 25]. In an attempt to make it generalizable across institutions, a broad free-text, natural language search criteria were included. Which is supported by prior evidence to the applicability of similar searches in the literature [13]. The current study only searched through pre-specified but limited notes sections, and it may be incomplete. However, it is anticipated that inaccuracies affect only a very small proportion of the study cohort, as the electronic search performed with sensitivities and specificities close to 100% when compared to manual review. Lastly, the computable phenotype data acquisition is limited by the timing of notes writing and databases updates. Thus the role of computable phenotype in real-time use is work in progress. As variety of rapid data is coming out in addition to data from electronic medical records [26]. To enhance machine learning and artificial intelligence capabilities, we must be able to aggregate medical records data in timely fashion [27]. Our search strategy may help expedite this effort.

## Conclusion

This study details the derivation and validation of computable phenotype derivation and validation for ADHF in hospitalized patients. This can be widely adopted to improve the efficiency and accuracy of clinical research, aid the institutional assessment of ADHF outcomes and direct quality improvement projects.

## Supplementary information

**Additional file 1.** Identification of Study Population and Automated Electronic Data Extraction Strategy for ADHF. Details of patient selection and data extraction strategy.

**Additional file 2.** Search terms for ADHF. Details of Inclusion and Exclusion terms for ADHF.

**Additional file 3.** ICD-9 codes for Heart Failure and Acute Heart failure- (By excluding Chronic Heart failure codes). Tables of ICD-9 codes for Heart Failure and Acute Heart failure- (By excluding Chronic Heart failure codes).

## Data Availability

Not applicable.

## Abbreviations

ACE: Advanced Cohort Explorer
ADHF: Acute decompensated heart failure
EHR: Electronic health record
HF: Heart failure
ICD-9: International Classification of Diseases, Ninth Revision
UDP: United Data Platform
