# Supplementary material for: Derivation and validation of a computable phenotype for acute decompensated heart failure in hospitalized patients
Source: BMC Med Inform Decis Mak. 2020 May 7;20:85. doi: 10.1186/s12911-020-1092-5 (PMC7206747; doi:10.1186/s12911-020-1092-5)
Supplement: Supplementary file 1 — Additional file 1. Identification of Study Population and Automated Electronic Data Extraction Strategy for ADHF. Details of patient selection and data extraction strategy. [file 12911_2020_1092_MOESM1_ESM.docx]

**Additional File 1**

*Identification of Study Population*

Patient hospitalization data was extracted from the UDP using an ACE hospital admission query. A total of 473,146 hospital admits in 314,988 patients were found during the study period listed above. From the patient number of 314,988 there were 44,867 patients excluded for age less than 18 years, 13,678 patients excluded for no research authorization in accordance with the Minnesota Health Records Act (Minnesota Statue 144.291-144.298) that states only patients who consented research authorization of their medical record data can be included for study, and 256,443 patients included ad total eligible patients.

*Automated Electronic Data Extraction Strategy for ADHF*

To find patients with ADHF we first had to find patients with any heart failure. To do this a study author adept at building ACE electronic search queries constructed a query using ICD9 codes for heart failure (Listed in **additional file 3**). This query was then run on a random sample of 1000 study patients obtained from the hospital admit query. Manual interrogation of clinical notes of the ICD9 query ‘hits’ was done in first 355 patients to determine how ADHF was documented in the patient’s medical record. From this manual interrogation of the clinical notes of the ICD9 ‘hits’ a clinical note text query was created.

To further develop the electronic search query, all synonyms, abbreviations, medical acronyms, and keywords associated with ADHF were entered into an ACE text query. (Listed in **additional file 2**) Furthermore, a comprehensive list of terms to exclude was developed to make the electronic search algorithm more specific. For instance, phrases such as “prior,” “no history of,” “denies,” “rule out,” or “negative for” were excluded. To establish a more uniform methodology, the application of the automated algorithm to note searches was restricted to the Diagnosis section of the patient’s clinical note records. The automated electronic algorithm was further refined in 2 random series of 100 patients taken from the study population described above and validated in an independent random cohort of 100 patients. (**Figure 1**) The sensitivity, specificity, and positive and negative predictive values of the automated digital algorithm and International Classification of Diseases, Ninth Revision (ICD-9) codes were compared with comprehensive medical record review (reference standard) for the ADHF.
