# Supplementary material for: Derivation and validation of a computable phenotype for acute decompensated heart failure in hospitalized patients
Source: BMC Med Inform Decis Mak. 2020 May 7;20:85. doi: 10.1186/s12911-020-1092-5 (PMC7206747; doi:10.1186/s12911-020-1092-5)
Supplement: Supplementary file 2 — Additional file 2. Search terms for ADHF. Details of Inclusion and Exclusion terms for ADHF. [file 12911_2020_1092_MOESM2_ESM.docx]

**Additional File 2.**

**Search terms for ADHF**

*Inclusion terms for ADHF*

*“Acute heart failure* ***OR*** *acute on chronic biventricular failure* ***OR*** *acute on chronic heart failure* ***OR*** *acute on chronic CHF* ***OR*** *acute on chronic HF* ***OR*** *acute on chronic CCF* ***OR*** *acute on chronic Congestive cardiac failure* ***OR*** *acute on chronic Congestive heart failure* ***OR*** *acute bi-ventricular heart failure* ***OR*** *acute biventricular heart failure* ***OR*** *end-stage ischemic cardiomyopathy with biventricular* ***OR*** *end-stage bi-ventricular heart failure* ***OR*** *worsening of heart failure* ***OR*** *worsening of CHF* ***OR*** *worsening of HF* ***OR*** *worsening of CCF* ***OR*** *worsening of Congestive cardiac failure* ***OR*** *worsening of Congestive heart failure* ***OR*** *worsening of biventricular failure* ***OR*** *exacerbation of heart failure* ***OR*** *exacerbation of CHF* ***OR*** *exacerbation of HF* ***OR*** *exacerbation of CCF* ***OR*** *exacerbation of Congestive cardiac failure* ***OR*** *exacerbation of Congestive heart failure* ***OR*** *exacerbation of biventricular failure* ***OR*** *decompensated heart failure* ***OR*** *Decompensated CHF* ***OR*** *decompensated HF* ***OR*** *decompensated CCF* ***OR*** *decompensated Congestive cardiac failure* ***OR*** *decompensated Congestive heart failure* ***OR*** *decompensated biventricular failure* ***OR*** *acute diastolic heart failure* ***OR*** *acute systolic heart failure* ***OR*** *acute diastolic congestive heart failure* ***OR*** *acute systolic congestive heart failure* ***OR*** *CHF exacerbation* ***OR*** *congestive heart failure exacerbation* ***OR*** *CCF exacerbation* ***OR*** *HF exacerbation* ***OR*** *heart failure exacerbation* ***OR*** *worsening CHF* ***OR*** *worsening CCF* ***OR*** *worsening congestive heart failure* ***OR*** *worsening heart failure* ***OR*** *worsening biventricular heart failure* ***OR*** *worsening congestive cardiac failure* ***OR*** *decompensated diastolic heart failure* ***OR*** *decompensated systolic heart failure* ***OR*** *worsening of his congestive heart failure* ***OR*** *worsening of her congestive heart failure”*

| **ICD-9 codes for Heart Failure** |
| --- |
| 428.0 Congestive heart failure, unspecified |
| 428.1 Left heart failure |
| 428.20 Systolic heart failure, unspecified |
| 428.21 Systolic heart failure, acute |
| 428.22 Systolic heart failure, chronic |
| 428.23 Systolic heart failure, acute on chronic |
| 428.30 Diastolic heart failure, unspecified |
| 428.31 Diastolic heart failure, acute |
| 428.32 Diastolic heart failure, chronic |
| 428.33 Diastolic heart failure, acute on chronic |
| 428.40 Combined systolic and diastolic heart failure, unspecified |
| 428.41 Combined systolic and diastolic heart failure, acute |
| 428.42 Combined systolic and diastolic heart failure, chronic |
| 428.43 Combined systolic and diastolic heart failure, acute on chronic |
| 428.9 Heart failure, unspecified |

*Exclusion terms for ADHF Within Sections: - Diagnosis- Principle Diagnosis, - Secondary Diagnoses,- Chief Complaint/Reason for Visit,- Brief Hospital Course*

*“COPD exacerbation* ***OR*** *chronic biventricular* ***OR*** *infectious exacerbation* ***OR*** *history of CHF exacerbation* ***OR*** *for signs of decompensated heart failure”*
