# Supplementary material for: Derivation and validation of a computable phenotype for acute decompensated heart failure in hospitalized patients
Source: BMC Med Inform Decis Mak. 2020 May 7;20:85. doi: 10.1186/s12911-020-1092-5 (PMC7206747; doi:10.1186/s12911-020-1092-5)
Supplement: Supplementary file 3 — Additional file 3. ICD-9 codes for Heart Failure and Acute Heart failure- (By excluding Chronic Heart failure codes). Tables of ICD-9 codes for Heart Failure and Acute Heart failure- (By excluding Chronic Heart failure codes). [file 12911_2020_1092_MOESM3_ESM.docx]

**Additional File 3.**

| **ICD 9 codes for Acute Heart failure- (By excluding Chronic Heart failure codes)** |
| --- |
| 428.1 Left heart failure |
| 428.21 Systolic heart failure, acute |
| 428.23 Systolic heart failure, acute on chronic |
| 428.31 Diastolic heart failure, acute |
| 428.33 Diastolic heart failure, acute on chronic |
| 428.41 Combined systolic and diastolic heart failure, acute |
| 428.43 Combined systolic and diastolic heart failure, acute on chronic |
